# Genetic polymorphisms of pharmacogenomic VIP variants in the Mongol of Northwestern China

**DOI:** 10.1186/s12863-016-0379-0

**Published:** 2016-05-28

**Authors:** Tianbo Jin, Xugang Shi, Li Wang, Huijuan Wang, Tian Feng, Longli Kang

**Affiliations:** Key Laboratory of High Altitude Environment and Genes Related to Diseases of Tibet Autonomous Region, School of Medicine, Xizang Minzu University, Xianyang, 712082 China; Key Laboratory for Basic Life Science Research of Tibet Autonomous Region, School of Medicine, Xizang Minzu University, #6 East Wenhui Road, Xianyang, 712082 Shaanxi China; National Engineering Research Center for Miniaturized Detection Systems, Xi’an, 710069 China; School of Life Sciences, Northwest University, Xi’an, Shaanxi 710069 China

**Keywords:** Pharmacogenomics, Genetic polymorphisms, Mongol, VIP variant

## Abstract

**Background:**

Within a population, the differences of pharmacogenomic variant frequencies may produce diversities in drug efficacy, safety, and the risk associated with adverse drug reactions. With the development of pharmacogenomics, widespread genetic research on drug metabolism has been conducted on major populations, but less is known about minorities.

**Results:**

In this study, we recruited 100 unrelated, healthy Mongol adults from Xinjiang and genotyped 85 VIP variants from the PharmGKB database. We compared our data with eleven populations listed in 1000 genomes project and HapMap database. We used χ^2^ tests to identify significantly different loci between these populations. We downloaded SNP allele frequencies from the ALlele FREquency Database to observe the global genetic variation distribution for these specific loci. And then we used Structure software to perform the genetic structure analysis of 12 populations.

**Conclusions:**

Our results demonstrated that different polymorphic allele frequencies exist between different nationalities,and indicated Mongol is most similar to Chinese populations, followed by JPT. This information on the Mongol population complements the existing pharmacogenomic data and provides a theoretical basis for screening and therapy in the different ethnic groups within Xinjiang.

## Background

It is well known that different individuals have different reactions to the same medications. Pharmacogenomics seeks to identify genetic markers that may influence a person’s response to pharmaceuticals. It will undoubtedly become an indispensable part of medical care in the future [1, 2]. Pharmacogenomic research seeks to identify single nucleotide polymorphisms (SNPs) or multiple gene signatures that are possibly associated with medication responses [3]. The goal of the research is to provide information for personalized medicine, i.e. give to the patient the optimal medication in optimal dose, and promote personalized therapeutics [4–6].

Numerous studies had shown that certain important genes and genetic variations affect critical functions during the drug reaction process. These genetic variations are called very important pharmacogenetic (VIP) variants and listed in the pharmacogenomics databases such as the Pharmacogenomics Knowledge Base (PharmGKB), the Pharmacogenetics of Membrane Transporters (PMT) database, and PharmaADME [6–8]. Currently, PharmGKB (http://www.pharmgkb.org) is the most comprehensive database and dedicates to propagating primary pharmacogenomic data and knowledge. They have extensively annotated the vital drug response genes and presented this information in VIP summaries, pathway diagrams, and curated literature [9].

In China, there are 56 different nationalities. Besides Han, the others account for approximately 100 million people. Due to the different genetic backgrounds and diverse environments of these minor populations, we distinguish them easily from the Han ethnicity. The Mongolian population represents one of the fifteen largest ethnic minorities in China [10]. They primarily live in the Inner Mongolia, Liaoning, Heilongjiang and the Xinjiang Uygur Autonomous Region. The areas are located in the grassland region of Northern China and significantly different with the Central Plains. Special living environments of the Mongol people shaped their unique gene distribution frequencies. An increasing number of studies suggest that genes related to drug response vary between different populations [11], so the pharmacogenomics population genetic studies of different population is valuable.

In this study, we random selected and genotyped 85 VIP variants from the PharmGKB VIP database in 100 Mongols from Xinjiang. We designed primers using MassARRAY Assay Design 3.0 Software [12]. We compared the Mongol’s allele frequencies with 11 populations from 1000 genomes project and the Mongol’s genotype frequencies and haplotype construction with 11 HapMap populations to identify the differences among them. The results will expand the current Mongol pharmacogenomic information and ethnic diversity. We aimed to provide new strategies for medical professionals through use genomic and molecular data to optimize drug administration and therapeutic treatment in the future.

## Methods

### Ethics statement

Blood samples and signed informed consent forms were obtained from all enrolls. All participants were informed both verbally and in writing of the procedures and purpose of the study, and signed informed consent documents. The clinical protocol was approved by the Clinical Research Ethics of Xizang Minzu University and Northwest University, and it is in compliance with Department of Health and Human Services (DHHS) regulations for human research subject protection.

### Study participants

We recruited 100 random unrelated Mongol adults (50 males and 50 females, average age range 25-40 years) from the Xinjiang Region of China and collected blood samples. The detailed recruitment criteria are the sample have good health body and had at least three generations of exclusive ethnic ancestries. They rarely communicate with other ethnics in Xinjiang because they are still nomads which living on relatively limited pasture. They were determined to be a representative Mongol population sample with regard to both ancestry and environmental exposure.

### Variant selection and genotyping

Using the PharmGKB database, we screened published genetic polymorphisms associated with VIP variants, and finally 85 genetic variant loci from 37 genes were randomly selected for our investigation. We extracted genomic DNA from whole blood using a GoldMag-Mini Whole Blood Genomic DNA Purification Kit (GoldMag Ltd. Xi’an, China) according to the manufacturer’s protocol. The genomic DNA concentration was measured by absorbance at 260 nm using a NanoDrop 2000C (Thermo Scientific, Waltham, Massachusetts, USA). We used the Sequenom MassARRAY Assay Design 3.0 software (San Diego, California, USA) to design multiplexed SNP MassEXTEND arrays [12]. We utilized a Sequenom MassARRAY RS1000 (San Diego, California, USA) to genotype the SNPs according to the manufacturer’s instructions. Sequenom Typer 4.0 Software was used for data collection and analysis as described previously [13].

### Statistical analyses

We used Microsoft Excel and the SPSS 19.0 statistical package (SPSS, Chicago, IL) to perform a Hardy–Weinberg Equilibrium (HWE) analysis and χ^2^ tests. All *p* values calculated were two-sided and Bonferroni’s multiple adjustment was used to correction. The values were considered statistically significant when *p* < 0.05 and *p* < 0.05/(85 × 11), respectively [14]. We analyzed each variant frequency in Mongols using an exact test to identify those that departed from HWE. We downloaded the allele frequencies of 85 loci in eleven randomly population of 1000 genomes project, which are a population of African ancestry in the southwestern USA (ASW); a population of Chinese Dai in Xishuangbanna, China (CDX); a Utah residents population (CEPH) with North and Western European Ancestry (CEU); the Chinese Han in Beijing, China (CHB); the Gujarati Indians in Houston, Texas, USA (GIH); the Japanese population in Tokyo, Japan (JPT); the Luhya people in Webuye, Kenya (LWK); people of Mexican ancestry from Los Angeles, USA (MXL); a population of Puerto Ricans from Puerto Rico (PUR); the Tuscan people of Italy (TSI); and the Yoruba in Ibadan, Nigeria (YRI). We downloaded the genotype frequencies of 85 variation loci in eleven populations from the HapMap database that are ASW; a northwestern European population (CEU); CHB; a Chinese population of metropolitan Denver, Colorado, USA (CHD); GIH; JPT; LWK; people of Mexican ancestry living in Los Angeles, California, USA (MEX); the Maasai people in Kinyawa, Kenya (MKK); TSI; and YRI. We first compared the allele frequencies difference between Mongolian and 11 random 1000 genomes project popualtions and calculate the correlation coefficient (R^2^) among the minor different population, then compared and calculated the selected SNP’s variant frequencies between the Mongol people and eleven HapMap populations (data from the second phase of HapMap: http://hapmap.ncbi.nlm.nih.gov) using a χ^2^ test. Afterwards, we downloaded the SNP allele frequencies of selected loci from the ALlele FREquency Database (http://alfred.med.yale.edu, ALFRED) and analyzed the global genetic variation patterns. We used Haploview software package (4.2) to perform the linkage disequilibrium (LD) analysis constructed haplotype, and genetic association of significant polymorphism loci.

### Analysis of population genetic structures

There are studies proved that the center of study which research human origins, DNA forensics and complex diseases is population genetic structure. It is also important to our study as a pharmacogenomics population study. Structure analysis is common in population genetic study. To further investigate variation at the VIP locus in terms of population structure we used STRUCTURE ver. 2.3.1 (Pritchard Lab, Stanford University,USA, http://pritchardlab.stanford.edu/structure.html) which based on the Bayesian clustering algorithmto assign the samples within a hypothetical K number of populations hypothesized by Pritchard et al [15]. We performed structure analysis using ancestry model with correlated allele frequencies among clusters. K = 2 to 8 is the range of possible numbers of clusters and 12 trials were run for each K. We performed the MCMC analyses for each structure analysis was run for 10,000 after an initial burn-in period of 10,000 for data collection. △K was calculated to identified the most likely number of clusters by STRUCTURE HARVESTER [16].

## Results

**Fig. 1:**
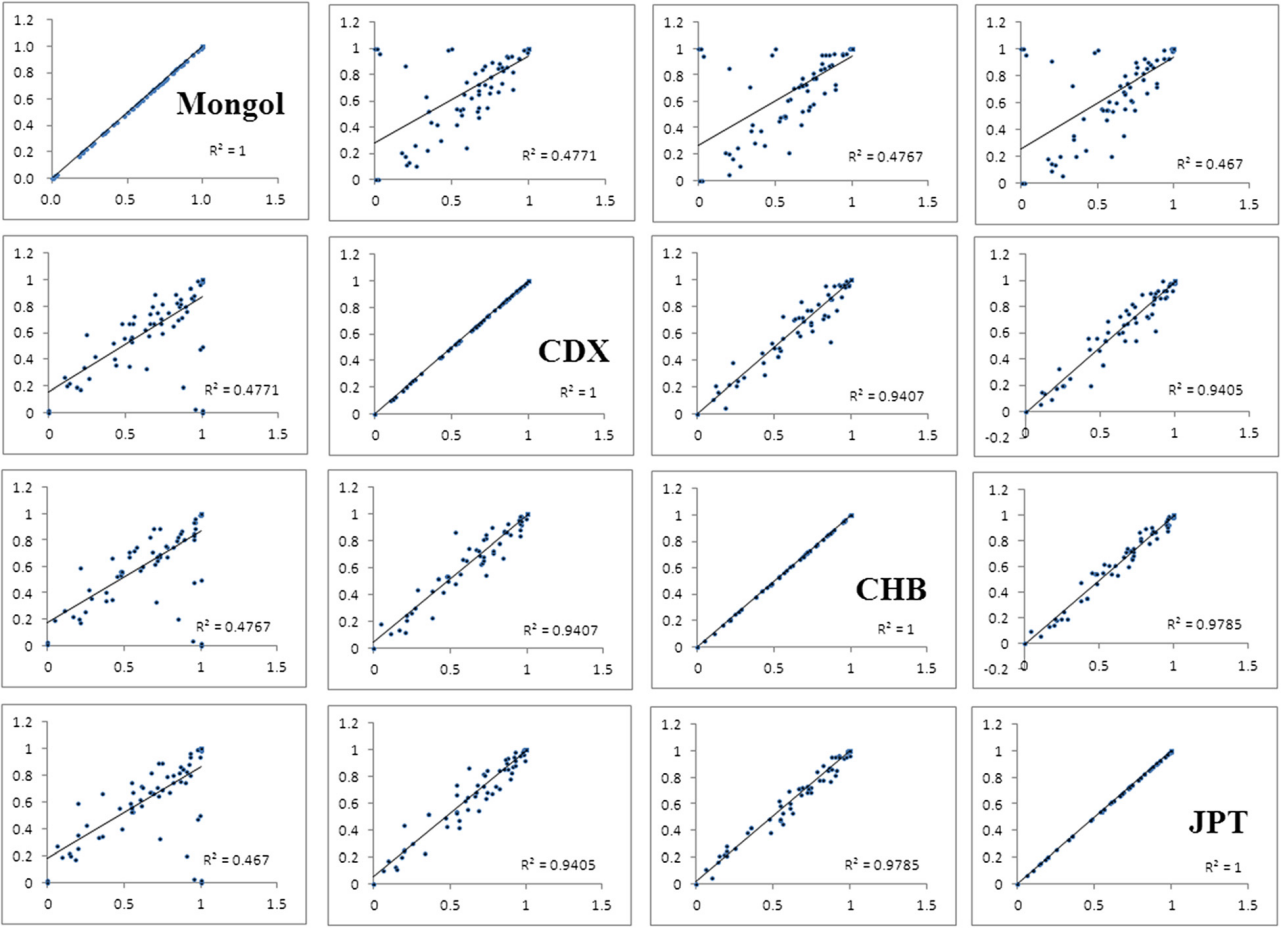
Pairwise comparisons of difference in correlation coefficient value R^2^

We sequenced 85 VIP variants from 100 Mongols. The selected SNP PCR primers were designed using the Sequenom MassARRAY Assay Design 3.0 Software. Information regarding the selected VIP loci and their genotype frequencies is listed in Table 1, including the genes, their positions, the nucleotide change, the amino acid translation, the calculated allele frequencies, and the genotype frequencies for Mongols. Several variants, such as rs698, rs1695, rs5219, rs16974, rs20417, rs890293, rs2740574, and rs3211371, did not meet HWE with a 5 % significance level and were not included in the final 85 loci analyzed. We first compared the allele frequencies differences among the Mongols and the selected 11 groups from 1000 genomes project database (*p* < 0.05). We found that there are some loci have significantly different between them. In ASW population, there are 22 loci exist different with Mongol. The results of other groups are as follows: CDX, 14; CEU, 19; CHB,15; GIH,15; JPT,15; LWK,18; MXL,18; PUR, 22; TSI, 18; YRI, 18(Table 2), respectively. In Fig. 1, we selected CDX, CHB and JPT which are the minimum difference population compared with Mongol population to calculate the correlation coefficient, R^2^. From the allele frequencies difference comparison, we figure out one initial conclusion that the Mongolian is relatively close to CDX, followed by CHB and JPT.

**Table 1:** Basic characteristics of the selected VIP variants from the PharmGKB database

SNP ID	Genes	Position	Chr	Categories		Alleles		Amino Acid Translation	Mongol		
				Family	Phase	A	B		AA	AB	BB
rs1801131	MTHFR	11854476	1	Methylenetetrahydrofolate reductase family	Phase I	C	A	Glu429Ala	8	34	58
rs1801133	MTHFR	11856378	1		Phase I	T	C	Ala222Val	9	39	52
rs890293	CYP2J2	60392494	1	Cytochrome P450 superfamily	Phase I	G	T		0	95	5
rs3918290	DPYD	97915614	1	-	PhaseI	G	A		100	0	0
rs6025	F5	169519049	1	-	Others	G	A	Arg534Gln	99	1	0
rs20417	PTGS2	186650321	1	-	Phase I	G	C		97	0	3
rs689466	PTGS2	186650751	1	-	Phase I	A	G		45	45	10
rs4124874	UGT1A1	234665659	2	UDP-glucuronosyltransferase family	Phase II	C	A		10	46	44
rs10929302	UGT1A1	234665782	2		Phase II	G	A		65	30	5
rs4148323	UGT1A1	234669144	2		Phase II	A	G	Gly71Arg	7	34	59
rs7626962	SCN5A	38620907	3	Sodium channel gene family	Others	G	T	Ser1103Tyr	100	0	0
rs1805124	SCN5A	38645420	3		Others	G	A	Pro1090Leu	1	25	74
rs6791924	SCN5A	38674699	3		Others	G	A	Arg34Cys	100	0	0
rs3814055	NR1I2	119500035	3	Nuclear receptor family	Others	C	T		46	47	7
rs2046934	P2RY12	151057642	3	G-protein coupled receptor family	Others	T	C		63	34	3
rs1065776	P2RY1	152553628	3		Others	T	C	Ala19Ala	0	7	93
rs701265	P2RY1	152554357	3		Others	G	A	Val262Val	9	52	39
rs975833	ADH1A	100201739	4	Alcohol dehydrogenase family	Phase I	G	C		33	52	15
rs2066702	ADH1B	100229017	4		Phase I	C	T	Arg370Cys	100	0	0
rs1229984	ADH1B	100239319	4		Phase I	G	A	His48Arg	45	44	11
rs698	ADH1C	100260789	4		Phase I	A	G	Ile350Val	67	25	7
rs17244841	HMGCR	74642855	5	-	Phase I	A	T		99	1	0
rs3846662	HMGCR	74651084	5	-	Phase I	T	C		30	52	18
rs17238540	HMGCR	74655498	5	-	Phase I	T	G		100	0	0
rs1042713	ADRB2	148206440	5	Adrenergic receptors family	Phase I	G	A	Arg16Gly	26	53	21
rs1042714	ADRB2	148206473	5		Phase I	G	C	Gln27Glu	6	42	52
rs1800888	ADRB2	148206885	5		Phase I	C	T	Thr164Ile	99	0	0
rs1142345	TPMT	18130918	6	Methyltransferase superfamily	Phase II	G	A	Tyr240Cys	0	2	98
rs1800460	TPMT	18139228	6		Phase II	A	G	Ala154Thr	0	1	99
rs2066853	AHR	17379110	7	-	Others	G	A	Arg554Lys	35	48	16
rs1045642	ABCB1	87138645	7	ATP-binding cassette (ABC) transporters superfamily	Others	T	C	Ile1145Ile	17	47	36
rs2032582	ABCB1	87160618	7		Others	G	T	Ser893Ala	25	35	15
rs2032582	ABCB1	87160618	7		Others	G	A	Ser893Thr	25	10	2
rs2032582	ABCB1	87160618	7		Others	T	A		15	13	2
rs1128503	ABCB1	87179601	7		Others	T	C	Gly412Gly	37	48	13
rs10264272	CYP3A5	99262835	7	Cytochrome P450 superfamily	Phase I	C	T	Lys208Lys	100	0	0
rs776746	CYP3A5	99270539	7		Phase I	G	A		79	20	1
rs4986913	CYP3A4	99358459	7		Phase I	C	T	Pro467Ser	100	0	0
rs4986910	CYP3A4	99358524	7		Phase I	T	C	Met445Thr	100	0	0
rs4986909	CYP3A4	99359670	7		Phase I	C	T	Pro416Leu	100	0	0
rs12721634	CYP3A4	99381661	7		Phase I	T	C	Leu15Pro	100	0	0
rs2740574	CYP3A4	99382096	7		Phase I	A	G		97	2	1
rs3815459	KCNH2	150644394	7	Eag family	Others	A	G		40	48	12
rs36210421	KCNH2	150644428	7		Others	G	T	Arg707Leu	100	0	0
rs12720441	KCNH2	150647304	7		Others	C	T	Arg444Trp	100	0	0
rs3807375	KCNH2	150667210	7		Others	A	G		56	37	7
rs4986893	CYP2C19	96540410	10	Cytochrome P450 superfamily	Phase I	G	A	Trp212null	88	11	1
rs4244285	CYP2C19	96541616	10		Phase I	G	A	Pro227Pro	69	26	5
rs1799853	CYP2C9	96702047	10		Phase I	C	T	Arg144Cys	100	0	0
rs1801252	ADRB1	115804036	10	Adrenergic receptors family	Phase I	G	A	Ser49Gly	5	26	69
rs1801253	ADRB1	115805056	10		Phase I	C	G	Gly389Arg	69	26	4
rs5219	KCNJ11	17409572	11	Inward-rectifier potassium channel family	Others	C	T	Lys23Glu	39	54	7
rs1695	GSTP1	67352689	11	Glutathione S-transferase family	Phase II	A	G	Ile105Val	52	46	2
rs1138272	GSTP1	67353579	11		Phase II	T	C	Ala114Val	0	3	97
rs1800497	ANKK1	113270828	11	Ser/Thr protein kinase family	Phase I	T	C	Glu713Lys	7	40	51
rs6277	DRD2	113283459	11	G-protein coupled receptor family	Others	C	T	Pro290Pro	79	19	2
rs4149056	SLCO1B1	21331549	12	Solute carrier family	Others	T	C	Val174Ala	71	28	1
rs7975232	VDR	48238837	12	Nuclear receptor family	Others	C	A		42	51	7
rs1544410	VDR	48239835	12		Others	G	A		72	26	2
rs2239185	VDR	48244559	12		Others	T	C		7	51	42
rs1540339	VDR	48257326	12		Others	G	A		18	49	33
rs2239179	VDR	48257766	12		Others	A	G		46	43	11
rs3782905	VDR	48266167	12		Others	C	G		62	35	2
rs2228570	VDR	48272895	12		Others	T	C	Met51Arg, Met51Lys, Met51Thr	9	51	40
rs10735810	VDR	48272895	12		Others	C	T		38	40	9
rs11568820	VDR	48302545	12		Others	G	A		55	36	7
rs1801030	SULT1A1	28617485	16	Sulfotransferase family	Phase II	A	G	Val223Met	100	0	0
rs3760091	SULT1A1	28620800	16		Phase II	C	G		32	43	18
rs7294	VKORC1	31102321	16	-	Phase I	G	A		67	32	1
rs9934438	VKORC1	31104878	16	-	Phase I	G	A		2	35	63
rs28399454	CYP2A6	41351267	19	Cytochrome P450 superfamily	Phase I	G	A	Val365Met	100	0	0
rs28399444	CYP2A6	41354190	19		Phase I	AA	-	Glu197Ser, Glu197Arg	100	0	0
rs1801272	CYP2A6	41354533	19		Phase I	T	A	Leu160His	95	3	0
rs28399433	CYP2A6	41356379	19		Phase I	G	T		1	20	79
rs3745274	CYP2B6	41512841	19		Phase I	G	T	Gln172His	65	29	6
rs28399499	CYP2B6	41518221	19		Phase I	T	C	Ile328Thr	99	1	0
rs3211371	CYP2B6	41522715	19		Phase I	C	T	Arg487Cys	0	100	0
rs12659	SLC19A1	46951556	21	Solute carrier family	Others	C	T	Pro192Pro	19	51	30
rs1051266	SLC19A1	46957794	21		Others	G	A	His27Arg	18	50	31
rs1131596	SLC19A1	46957916	21		Others	T	C		19	38	24
rs4680	COMT	19951271	22	-	Phase II	A	G	Val158Met	10	38	52
rs59421388	CYP2D6	42523610	22	Cytochrome P450 superfamily	Phase I	C	T	Val287Met	100	0	0
rs28371725	CYP2D6	42523805	22		Phase I	G	A		86	13	0
rs16947	CYP2D6	42523943	22		Phase I	G	A		44	35	0
rs61736512	CYP2D6	42525134	22		Phase I	C	A/G/T	Val136Met	100	0	0
rs28371706	CYP2D6	42525772	22		Phase I	C	T	Thr107Ile	100	0	0
rs5030656	CYP2D6	42524176:42524176	22		Phase I	AAG	-		100	0	0

**Table 2:** Significant VIP variants in Mongols compared with the eleven populations which selected from 1000 genomes project

SNP ID	*p* < 0.05										
	ASW	CDX	CEU	CHB	GIH	JPT	LWK	MXL	PUR	TSI	YRI
rs10264272	***1.42E-05***	-	-	-	-	-	1.01E-01	***1.35E-10***	***3.35E-06***	***7.27E-48***	***3.30E-02***
rs1042713	1.90E-01	2.05E-01	2.09E-01	1.90E-01	1.74E-01	1.70E-01	1.63E-01	1.57E-01	1.84E-01	2.00E-01	1.83E-01
rs1042714	1.29E-01	1.31E-01	2.37E-01	1.30E-01	1.17E-01	1.32E-01	1.19E-01	1.27E-01	1.95E-01	1.87E-01	1.29E-01
rs1045642	2.25E-01	1.59E-01	2.35E-01	1.58E-01	2.36E-01	1.88E-01	2.43E-01	1.86E-01	1.61E-01	1.81E-01	2.49E-01
rs1051266	1.54E-01	1.67E-01	2.22E-01	1.98E-01	2.41E-01	1.64E-01	2.01E-01	2.65E-01	2.20E-01	2.12E-01	1.93E-01
rs1065776	7.08E-02	***1.10E-05***	***8.68E-04***	***1.09E-04***	***8.98E-03***	***2.49E-03***	7.70E-02	***4.22E-04***	***4.99E-03***	***9.58E-05***	8.60E-02
rs10735810	4.49E-01	2.18E-01	2.79E-01	2.83E-01	3.77E-01	3.30E-01	4.69E-01	2.42E-01	3.16E-01	3.10E-01	4.55E-01
rs10929302	1.62E-01	7.71E-02	1.47E-01	7.55E-02	2.47E-01	7.73E-02	1.85E-01	1.74E-01	1.61E-01	1.00E-01	1.87E-01
rs1128503	4.27E-01	1.45E-01	2.53E-01	1.68E-01	1.73E-01	1.56E-01	4.73E-01	2.29E-01	2.69E-01	2.59E-01	4.48E-01
rs1131596	1.94E-01	1.59E-01	2.04E-01	1.81E-01	2.21E-01	1.61E-01	2.56E-01	2.42E-01	2.00E-01	1.94E-01	2.37E-01
rs1138272	***4.98E-07***	***6.87E-16***	***4.54E-03***	***6.87E-16***	***2.20E-03***	***6.87E-16***	***6.75E-09***	***2.13E-04***	***6.74E-06***	***1.43E-04***	***6.87E-16***
rs1142345	***4.41E-03***	***1.55E-06***	***8.22E-07***	***2.81E-16***	***7.94E-08***	***5.19E-09***	***9.34E-03***	***4.20E-05***	***3.05E-03***	***9.93E-11***	***2.63E-04***
rs11568820	4.50E-01	1.56E-01	1.09E-01	2.10E-01	1.89E-01	2.38E-01	5.72E-01	1.15E-01	1.13E-01	1.06E-01	6.86E-01
rs1229984	2.02E-01	3.25E-01	2.00E-01	3.79E-01	1.98E-01	3.96E-01	2.02E-01	1.88E-01	1.92E-01	1.94E-01	2.02E-01
rs12659	2.31E-01	1.65E-01	2.21E-01	1.88E-01	2.51E-01	1.60E-01	1.66E-01	2.62E-01	2.19E-01	2.09E-01	1.90E-01
rs12720441	1.00E + 00	1.00E + 00	1.00E + 00	1.00E + 00	1.00E + 00	1.00E + 00	1.00E + 00	1.00E + 00	1.00E + 00	1.00E + 00	1.00E + 00
rs1540339	3.35E-01	1.98E-01	2.90E-01	2.11E-01	2.72E-01	2.17E-01	4.10E-01	2.53E-01	2.82E-01	2.69E-01	3.65E-01
rs1544410	1.27E-01	***3.56E-02***	2.98E-01	***3.26E-02***	2.59E-01	***4.48E-02***	1.13E-01	7.41E-02	2.43E-01	2.49E-01	1.47E-01
rs16947	2.23E-01	9.14E-02	1.54E-01	9.11E-02	2.41E-01	9.15E-02	4.12E-01	1.10E-01	2.01E-01	1.90E-01	3.38E-01
rs1695	2.42E-01	1.06E-01	1.95E-01	1.09E-01	1.39E-01	1.14E-01	2.81E-01	3.22E-01	1.79E-01	1.29E-01	1.98E-01
rs17238540	***3.20E-03***	-	***1.07E-15***	-	-	-	***2.03E-03***	***9.22E-07***	***1.14E-12***	***6.91E-08***	***2.99E-03***
rs17244841	***3.79E-03***	***4.44E-45***	***2.57E-12***	***4.44E-45***	***4.44E-45***	***1.82E-10***	***1.81E-03***	***3.30E-06***	***8.88E-13***	***4.29E-07***	***2.75E-03***
rs1799853	***1.71E-06***	-	***2.34E-02***	-	***1.22E-05***	-	-	***3.82E-03***	***1.69E-02***	***2.50E-02***	-
rs1800460	***9.41E-09***	***4.44E-45***	***1.66E-07***	***4.44E-45***	***4.44E-45***	***4.44E-45***	***4.44E-45***	***3.30E-06***	***2.57E-05***	***5.05E-13***	***4.44E-45***
rs1800497	2.14E-01	2.22E-01	1.24E-01	2.16E-01	1.20E-01	1.84E-01	1.77E-01	2.16E-01	1.19E-01	1.22E-01	1.86E-01
rs1800888	-	-	***1.07E-15***	-	-	-	-	-	-	***6.14E-17***	-
rs1801131	1.07E-01	1.26E-01	1.51E-01	1.05E-01	2.12E-01	1.10E-01	1.09E-01	1.11E-01	1.06E-01	1.41E-01	1.13E-01
rs1801133	1.40E-01	1.40E-01	1.25E-01	2.32E-01	1.38E-01	1.74E-01	1.46E-01	2.34E-01	2.19E-01	2.33E-01	1.43E-01
rs1801252	6.55E-02	6.41E-02	6.28E-02	6.45E-02	6.35E-02	6.43E-02	1.47E-01	1.15E-01	8.73E-02	5.96E-02	8.24E-02
rs1801253	2.01E-01	8.00E-02	1.68E-01	8.58E-02	9.53E-02	6.70E-02	1.53E-01	6.09E-02	9.70E-02	1.82E-01	2.94E-01
rs1801272	9.73E-01	9.83E-01	9.43E-01	9.83E-01	9.72E-01	9.83E-01	9.83E-01	9.65E-01	9.77E-01	9.30E-01	9.83E-01
rs1805124	1.22E-01	***2.93E-02***	6.49E-02	***3.75E-02***	7.93E-02	***3.85E-02***	1.55E-01	5.42E-02	1.20E-01	1.00E-01	1.77E-01
rs20417	6.15E-01	9.18E-01	7.73E-01	9.06E-01	7.81E-01	9.17E-01	6.48E-01	7.29E-01	7.28E-01	8.22E-01	5.42E-01
rs2046934	7.68E-02	7.56E-02	7.82E-02	7.93E-02	7.48E-02	7.71E-02	7.48E-02	7.54E-02	7.44E-02	7.61E-02	7.73E-02
rs2066702	6.37E-02	-	-	-	-	-	***1.79E-02***	***3.41E-08***	***2.32E-10***	-	1.45E-01
rs2066853	1.69E-01	1.99E-01	2.60E-01	1.58E-01	2.51E-01	1.79E-01	1.94E-01	2.45E-01	2.39E-01	2.58E-01	1.75E-01
rs2228570	1.75E-01	2.40E-01	1.84E-01	1.80E-01	1.51E-01	1.43E-01	1.82E-01	2.17E-01	1.53E-01	1.58E-01	1.77E-01
rs2239179	1.37E-01	1.44E-01	2.39E-01	1.54E-01	2.39E-01	1.59E-01	1.47E-01	1.38E-01	1.99E-01	1.80E-01	1.39E-01
rs2239185	2.69E-01	1.33E-01	3.05E-01	1.41E-01	2.42E-01	1.32E-01	3.30E-01	1.82E-01	2.94E-01	2.96E-01	2.92E-01
rs2740574	6.05E-01	***3.29E-12***	***8.71E-08***	***3.29E-12***	***1.99E-03***	***3.29E-12***	7.90E-01	***1.22E-03***	5.35E-02	***4.58E-06***	7.11E-01
rs28371706	***2.11E-02***	-	-	-	-	-	5.24E-02	-	***3.96E-24***	-	1.14E-01
rs28371725	***7.01E-04***	***7.46E-03***	***2.18E-02***	***1.65E-03***	***3.43E-02***	***3.16E-04***	***1.41E-03***	***6.68E-04***	***2.13E-02***	***3.39E-02***	***4.43E-04***
rs28399433	***2.33E-02***	6.01E-02	***1.44E-02***	1.27E-01	6.80E-02	1.37E-01	***2.10E-02***	***2.26E-02***	***2.25E-02***	***1.69E-02***	***2.27E-02***
rs28399454	***5.13E-04***	-	-	-	-	-	***4.87E-05***	-	***3.96E-24***	-	***1.45E-02***
rs28399499	***3.79E-03***	***4.44E-45***	***4.44E-45***	***4.44E-45***	***4.44E-45***	***4.44E-45***	***1.77E-04***	***9.37E-19***	***1.82E-10***	***4.44E-45***	***8.27E-03***
rs3211371	3.94E-01	4.14E-01	3.57E-01	4.14E-01	3.56E-01	4.08E-01	4.14E-01	3.63E-01	3.57E-01	3.50E-01	4.08E-01
rs36210421	-	-	***6.00E-06***	-	***5.70E-46***	***6.74E-47***	-	***2.15E-29***	-	***6.14E-17***	-
rs3745274	1.79E-01	1.61E-01	1.26E-01	8.04E-02	2.18E-01	8.94E-02	1.83E-01	1.50E-01	1.74E-01	1.43E-01	2.17E-01
rs3760091	1.69E-01	1.79E-01	1.60E-01	1.64E-01	2.31E-01	1.65E-01	1.82E-01	1.78E-01	1.68E-01	1.85E-01	1.79E-01
rs3782905	5.54E-01	6.44E-01	4.26E-01	6.28E-01	5.24E-01	6.82E-01	5.48E-01	5.66E-01	4.68E-01	4.46E-01	5.58E-01
rs3807375	1.14E-01	1.07E-01	3.91E-01	1.06E-01	3.59E-01	1.11E-01	1.11E-01	2.19E-01	3.31E-01	3.97E-01	1.08E-01
rs3814055	1.27E-01	1.53E-01	1.43E-01	1.31E-01	1.91E-01	1.35E-01	1.25E-01	1.42E-01	1.89E-01	1.56E-01	1.31E-01
rs3815459	2.70E-01	1.82E-01	4.25E-01	1.61E-01	3.22E-01	1.87E-01	2.89E-01	2.83E-01	3.54E-01	3.97E-01	3.27E-01
rs3846662	3.96E-01	1.86E-01	1.60E-01	1.90E-01	2.68E-01	1.97E-01	4.66E-01	1.55E-01	1.85E-01	1.58E-01	4.58E-01
rs3918290	***5.03E-28***	-	***3.18E-44***	-	***2.72E-16***	-	-	-	-	***7.27E-48***	-
rs4124874	4.33E-01	2.03E-01	1.92E-01	1.47E-01	3.05E-01	1.37E-01	5.27E-01	2.44E-01	2.24E-01	1.98E-01	5.38E-01
rs4148323	9.94E-02	1.05E-01	9.82E-02	9.89E-02	1.01E-01	1.05E-01	9.82E-02	1.01E-01	9.82E-02	9.82E-02	9.82E-02
rs4149056	***3.68E-02***	***4.68E-02***	***4.74E-02***	***4.64E-02***	***2.73E-02***	***4.48E-02***	***2.74E-02***	***3.90E-02***	***4.48E-02***	8.70E-02	***2.48E-02***
rs4244285	6.40E-02	1.19E-01	6.35E-02	1.72E-01	1.68E-01	1.62E-01	8.47E-02	6.31E-02	6.34E-02	6.01E-02	6.53E-02
rs4680	1.23E-01	1.23E-01	2.29E-01	1.34E-01	2.10E-01	1.20E-01	1.20E-01	1.85E-01	1.85E-01	1.54E-01	1.54E-01
rs4986893	***1.93E-04***	***6.14E-03***	***1.93E-04***	***2.27E-03***	***2.96E-04***	***5.46E-03***	***4.39E-04***	***1.93E-04***	***1.93E-04***	***1.93E-04***	***1.93E-04***
rs4986910	***5.03E-28***	-	***1.07E-15***	-	-	-	-	-	***6.74E-47***	-	-
rs4986913	-	-	-	-	***5.70E-46***	-	-	-	-	-	-
rs5219	1.85E-01	1.64E-01	1.59E-01	1.57E-01	1.79E-01	1.38E-01	2.13E-01	1.72E-01	1.49E-01	1.49E-01	2.14E-01
rs59421388	***1.71E-06***	-	-	-	-	-	***3.66E-02***	-	***6.74E-47***	-	***4.90E-03***
rs6025	***4.44E-45***	***4.44E-45***	***1.42E-08***	***4.44E-45***	***7.17E-24***	***4.44E-45***	***4.44E-45***	***9.37E-19***	***1.36E-16***	***2.56E-24***	***4.44E-45***
rs61736512	***1.71E-06***	-	-	-	-	-	***3.66E-02***	-	***6.74E-47***	-	***6.07E-03***
rs6277	***4.28E-02***	***1.69E-02***	3.46E-01	***1.42E-02***	1.87E-01	***2.09E-02***	***9.87E-03***	1.66E-01	2.93E-01	4.45E-01	***1.22E-02***
rs6791924	***6.55E-05***	-	-	-	-	-	***2.96E-02***	***1.35E-10***	***3.96E-24***	-	***1.66E-03***
rs689466	1.74E-01	2.50E-01	1.61E-01	2.17E-01	1.75E-01	1.99E-01	1.92E-01	1.47E-01	1.50E-01	1.60E-01	1.85E-01
rs698	7.48E-02	7.35E-02	2.80E-01	6.65E-02	1.29E-01	6.98E-02	7.49E-02	1.29E-01	1.91E-01	1.48E-01	6.94E-02
rs701265	3.38E-01	1.81E-01	1.87E-01	1.55E-01	1.76E-01	1.58E-01	4.38E-01	1.77E-01	1.65E-01	1.88E-01	4.39E-01
rs7294	2.93E-01	5.96E-02	1.57E-01	***4.55E-02***	4.69E-01	5.39E-02	2.53E-01	1.88E-01	1.79E-01	1.75E-01	3.28E-01
rs7626962	***6.55E-05***	-	-	-	-	-	***1.98E-08***	-	***1.68E-16***	-	***1.18E-03***
rs776746	5.43E-01	1.66E-01	***1.26E-02***	1.66E-01	1.36E-01	1.17E-01	7.44E-01	1.01E-01	1.25E-01	***1.45E-02***	6.95E-01
rs7975232	3.26E-01	1.42E-01	3.01E-01	1.41E-01	2.46E-01	1.37E-01	3.90E-01	1.77E-01	2.94E-01	2.96E-01	3.05E-01
rs890293	3.42E-01	4.34E-01	4.12E-01	4.16E-01	4.10E-01	4.29E-01	3.58E-01	4.24E-01	4.04E-01	4.11E-01	3.48E-01
rs975833	2.05E-01	3.46E-01	2.09E-01	3.72E-01	1.91E-01	3.80E-01	2.28E-01	2.50E-01	2.17E-01	1.99E-01	1.88E-01
rs9934438	6.28E-01	7.43E-02	3.64E-01	6.42E-02	6.01E-01	7.08E-02	7.79E-01	3.29E-01	3.99E-01	3.22E-01	7.49E-01

^**a**^Italics indicated that after adjustment *p <* 0.05 the locus has statistically significant

^**b**^The results has not the mathematics sense

We used χ^2^ analyses to compare differences in the variants’ genotype frequency distributions among the Mongols and eleven HapMap populations (without adjustment, *p* < 0.05; adjustment, *p* < 0.05/85 × 11). There were a number of loci had significantly different distribution frequencies among Mongols and the 11 HapMap populations that listed in Table 3 Without adjustment the ASW population had 20 different loci; CEU, 27; CHB, 19; CHD, 10; GIH, 24; JPT, 17; LWK, 24; MEX, 17; MKK, 22; TSI, 14; and YRI, 38. Once the data underwent multiple comparison adjustment, the numbers of significantly different loci variants were revised to 13, 13, 6, 1, 8, 5, 18, 3, 18, 6 and 26, respectively.

**Table 3:** Significant VIP variants in Mongols compared with the eleven HapMap populations after Bonferroni’s multiple adjustment

SNP ID	p < 0.05/(85 × 11)										
	ASW	CEU	CHB	CHD	GIH	JPT	LWK	MEX	MKK	TSI	YRI
rs10264272	-	-	-	-	-	-	***1.44532E-12*** ^***a***^	-^b^	***8.82E-08***	-	***8.95E-09***
rs1042713	0.27747316	0.04538453	0.20742869	0.12421983	0.47741241	0.58439074	0.54885721	0.90201422	0.32444254	0.01651791	0.32052007
rs1042714	-	8.50E-05	0.0164067	-	-	0.00044539	-	-	-	-	0.07513803
rs1045642	***4.92E-09***	0.00116183	0.54603257	0.29771668	0.00162633	0.28558754	-	0.45392674	***1.61E-08***	0.50240513	***2.41E-10***
rs1051266	0.2807523	0.01941254	0.33010486	0.12007109	0.00246504	0.95743171	0.00188424	0.00059997	***1.36E-05***	0.07011082	0.01298312
rs1065776	-	-	-	-	-	-	-	-	-	-	-
rs10735810	0.01884334	0.19825318	0.35306461	0.01687974	0.42789254	0.16219036	0.00044495	0.00959988	0.00592383	0.43490023	0.00575742
rs10929302	-	0.2213663	0.06968442	-	-	0.08008041	-	-	-	-	0.00749071
rs1128503	***3.62E-11***	0.00100994	0.18626034	0.47475329	0.86444934	0.72437343	***9.47E-20***	0.0231531	***1.73E-21***	0.00083246	***2.91E-21***
rs1131596	-	-	-	-	-	-	-	-	-	-	-
rs1138272	-	-	-	-	-	-	-	0.03123952	-	-	-
rs1142345	-	-	-	-	-	-	0.00012785	0.01252136	-	-	0.1353563
rs11568820	***8.07E-12***	0.39713354	0.00088999	0.06850479	0.00681425	0.00038384	***3.81E-24***	0.46841122	***8.28E-23***	0.75222572	***9.16E-37***
rs1229984	-	***1.20E-11***	***5.49E-10***	-	-	***7.68E-09***	-	-	-	-	***1.69E-11***
rs12659	-	-	-	-	-	-	-	-	-	-	-
rs12720441	-	-	-	-	-	-	-	-	-	-	-
rs12721634	-	-	-	-	-	-	-	-	-	-	-
rs1540339	***6.30E-07***	0.000116	0.02314118	0.00276558	***5.31E-05***	0.0048091	***1.48E-15***	0.01115187	***7.82E-16***	0.00014778	***1.52E-12***
rs1544410	0.06188271	***1.69E-08***	0.00273242	0.00242682	***3.33E-08***	0.39727054	0.04804346	0.14153371	***2.63E-06***	***2.71E-07***	0.00553292
rs16947	-	-	-	-	-	-	-	-	-	-	-
rs1695	0.00017138	0.0007169	0.21785447	0.42376541	0.07372284	6.16E-05	***2.60E-07***	***6.20E-08***	0.00864594	0.15360318	0.00135975
rs17238540	-	-	-	-	-	-	-	-	-	-	-
rs17244841	-	-	-	-	-	-	-	-	-	-	-
rs1799853	-	-	-	-	-	-	-	-	-	-	-
rs1800460	-	-	-	-	-	-	-	-	-	-	-
rs1800497	0.05635435	0.10573707	0.01289027	0.00481115	0.55312005	0.02909995	0.16955696	0.04539287	0.16542808	0.29503104	0.01419286
rs1800888	-	-	-	-	-	-	-	-	-	-	-
rs1801030	-	-	-	-	-	-	-	-	-	-	-
rs1801131	0.29838721	0.10171883	0.44379999	0.64433884	0.0183119	0.20392711	0.2737931	0.64086028	0.91251575	0.36340669	0.00184881
rs1801133	0.00034783	0.72955982	0.00135396	0.49992159	0.0319354	0.23529674	***1.68E-05***	0.05835697	***1.52E-07***	0.00349079	***4.10E-06***
rs1801252	-	-	0.0001403	-	-	0.0001681	-	-	-	-	***4.90E-05***
rs1801253	-	0.01885371	0.22338349	-	-	0.79204456	-	-	-	-	***2.70E-05***
rs1801272	-	***2.63E-33***	***1.46E-31***	-	-	***2.42E-30***	-	-	-	-	***4.91E-35***
rs1805124	0.02658648	0.36484631	0.54483156	0.22673707	0.35376474	0.6491555	0.00245978	0.86877609	***1.87E-06***	0.04053952	0.00013843
rs2032582	***2.07E-07***	0.46831684	0.02691764	0.0899833	0.00032517	0.11450139	***5.66E-17***	0.70811234	***6.28E-14***	0.96126181	-
rs2032582	-	-	-	-	-	-	-	-	-	-	-
rs2032582	-	-	-	-	-	-	-	-	-	-	-
rs20417	-	***1.04E-29***	***2.19E-29***	-	-	***3.82E-29***	-	-	-	-	***2.24E-24***
rs2046934	-	0.84184132	0.87995183	-	-	0.97034167	-	-	-	-	0.77148622
rs2066702	***1.06E-10***	-	-	-	-	-	***4.08E-07***	-	-	-	***5.65E-15***
rs2066853	0.65615202	***1.66E-11***	0.94214103	0.94774007	***2.18E-09***	0.49230972	0.2116787	***5.48E-06***	0.41238266	***9.43E-11***	0.62463192
rs2228570	-	-	-	-	-	-	-	-	-	-	-
rs2239179	0.19236008	0.00467976	0.16879391	0.0735274	0.00371014	0.07153574	***4.11E-08***	0.71139635	0.04517466	0.17315647	0.71742841
rs2239185	-	-	0.11123339	-	-	0.45274301	-	-	-	-	0.0001787
rs2740574	-	-	-	-	-	-	-	-	-	-	-
rs28371706	-	-	-	-	-	-	-	-	-	-	-
rs28371725	-	-	-	-	-	-	-	-	-	-	-
rs28399433	-	-	-	-	-	-	-	-	-	-	-
rs28399444	-	-	-	-	-	-	-	-	-	-	-
rs28399454	-	-	-	-	-	-	-	-	-	-	-
rs28399499	0.00193936	-	-	-	-	-	-	-	0.43590356	-	***9.04E-06***
rs3211371	-	-	-	-	-	-	-	-	-	-	-
rs36210421	-	-	-	-	-	-	-	-	-	-	-
rs3745274	0.12627303	0.2056118	0.70303194	0.34150665	0.00017843	0.84757978	0.04657355	0.04107996	0.00024687	0.1559596	***2.11E-05***
rs3760091	-	-	-	-	-	-	-	-	-	-	-
rs3782905	-	***4.47E-15***	***4.87E-18***	-	-	***1.49E-22***	-	-	-	-	***4.87E-19***
rs3807375	0.43616954	***2.01E-12***	0.86962796	0.83584673	***8.18E-11***	0.12181871	0.12372447	0.01752245	0.5934107	***2.36E-12***	0.57322879
rs3814055	0.83102212	0.52973507	0.45387793	0.20572098	0.03703383	0.18923006	0.29073405	0.7548396	0.05268438	0.26051487	0.09961755
rs3815459	-	-	0.3754275	-	-	0.00773141	-	-	-	-	***8.65E-06***
rs3846662	***8.13E-12***	0.6759055	0.19583564	0.0971148	0.00018701	0.15042586	***3.46E-24***	0.45840601	***2.29E-17***	0.72844286	***2.62E-26***
rs3918290	-	-	-	-	-	-	-	-	-	-	-
rs4124874	***1.52E-11***	0.0357679	0.20056719	0.8982781	***4.02E-07***	0.89376825	***3.20E-22***	0.00572364	***6.38E-23***	0.14836683	***3.23E-27***
rs4148323	-	***6.57E-08***	0.11727653	***1.15E-20***	***3.34E-08***	0.02782333	-	7.78E-05	-	-	***6.57E-08***
rs4149056	0.01202321	0.99516204	0.98870047	0.36757664	6.12E-05	0.19624085	***1.43E-05***	0.19712962	0.36973702	0.20946995	***1.34E-07***
rs4244285	-	0.75228568	0.00660126	-	-	0.09009154	-	-	-	-	0.73387157
rs4680	0.48647969	0.00082072	0.98587091	0.14463711	0.04170753	0.66470326	0.78746668	0.23399959	0.94982091	0.00298993	0.57419215
rs4986893	-	-	-	-	-	-	-	-	-	-	-
rs4986909	-	-	-	-	-	-	-	-	-	-	-
rs4986910	-	-	-	-	-	-	-	-	-	-	-
rs4986913	-	-	-	-	-	-	-	-	-	-	-
rs5030656	-	-	-	-	-	-	-	-	-	-	-
rs5219	-	-	-	-	-	-	-	-	-	-	-
rs59421388	-	-	-	-	-	-	-	-	-	-	-
rs6025	-	0.42877395	-	-	-	-	-	-	-	-	-
rs61736512	-	-	-	-	-	-	-	-	-	-	-
rs6277	-	***1.03E-13***	0.29571877	-	-	0.24782281	-	-	-	-	0.052309
rs6791924	-	-	-	-	-	-	-	-	-	-	-
rs689466	0.00012294	0.00040262	0.00388527	0.02658023	0.00036352	0.031278	***9.46E-12***	0.34461035	***1.46E-16***	0.00281474	***3.77E-08***
rs698	0.67209493	***3.15E-08***	0.00098605	0.00686794	0.18843861	0.00282244	0.51518149	0.14164572	0.31894381	0.05936975	0.00044968
rs701265	***1.10E-07***	0.00028496	0.22430801	0.08574165	0.01840946	0.09399066	***1.48E-16***	0.00740647	***6.36E-16***	***4.43E-05***	***3.39E-18***
rs7294	***3.00E-09***	***3.54E-05***	0.00145857	0.01140667	***2.58E-21***	0.1101385	***3.98E-08***	0.00317685	***1.58E-11***	0.00047099	***3.28E-12***
rs7626962	-	-	-	-	-	-	-	-	-	-	0.0012409
rs776746	***3.55E-17***	0.01253767	***5.28E-05***	0.00537234	0.00294358	0.00027677	***6.11E-31***	0.00270059	***2.59E-17***	0.10945504	***2.12E-34***
rs7975232	***5.21E-07***	***6.63E-07***	0.17255518	0.39866127	0.00031086	0.49657642	***1.18E-13***	0.06431886	***8.52E-13***	***3.91E-07***	***3.28E-09***
rs890293	-	-	-	-	-	-	-	-	-	-	-
rs975833	-	0.0999674	***5.65E-11***	-	-	***4.30E-09***	-	-	-	-	0.02956016
rs9934438	***1.86E-24***	***9.17E-14***	0.0001892	0.00092503	***1.98E-24***	0.02360989	***5.38E-33***	***1.64E-08***	***1.98E-32***	***1.07E-09***	***1.88E-41***

^a^ Italics indicated that after adjustment *p <* 0.05/(85*11) the locus has statistically significant

^b^ The results has not the mathematics sense

When p < 0.05, rs1540339 locus (46489G > A) which located in an intron region of *VDR* (1, 25- dihydroxyvitamin D3 receptor), showed the greatest number of significant differences between Mongol and 11 HapMap populations; the SNP rs776746 (12083G > A) is a SNP of CYP3A5 which located in an intron region and a significant locus that observed in these populations except TSI. After Bonferroni’s multiple adjustment (*p* < 0.05/(85 × 11)), the number of HapMap populations with a significantly different rs1540339 locus changed very large which included CEU, CHB, CHD, JPT, MEX and TRI. The rs776746 locus also changed very large which except TSI added CEU, CHD, GIH, JPT and MEX.

Of the 85 variants analyzed, 74 could be classified as part of a superfamily. When the gene superfamily categories were tallied, the number of the associated variants with significantly different frequencies between the Mongols and the eleven HapMap populations were as follows: ASW, 10; CEU, 9; CHB, 5; CHD, 1; GIH, 5; JPT, 4; LWK, 14; MEX, 1; MKK, 14; TSI, 4; and YRI, 21 (Table 4). A number of distinct loci were significantly different and included several pharmacogenomic superfamilies such as the nuclear receptor family, the sodium channel gene family, and the methylenetetrahydrofolate reductase family.

**Table 4:** The VIP variants in Mongols compared with eleven HapMap groups according to the gene superfamily classification

ASW	CEU	CHB	CHD	GIH	JPT	LWK	MEX	MKK	TSI	YRI
rs1045642	rs1229984	rs1229984	rs4148323	rs1540339	rs1229984	rs10264272	rs1695	rs10264272	rs1544410	rs10264272
rs1128503	rs1544410	rs1801272		rs1544410	rs1801272	rs1128503		rs1045642	rs3807375	rs1045642
rs11568820	rs1801272	rs3782905		rs3807375	rs3782905	rs11568820		rs1051266	rs701265	rs1128503
rs1540339	rs3782905	rs776746		rs4124874	rs975833	rs1540339		rs1128503	rs7975232	rs11568820
rs2032582	rs3807375	rs975833		rs4148323		rs1695		rs11568820		rs1229984
rs2066702	rs4148323					rs1801133		rs1540339		rs1540339
rs4124874	rs6277					rs2032582		rs1544410		rs1801133
rs701265	rs698					rs2066702		rs1801133		rs1801252
rs776746	rs7975232					rs2239179		rs1805124		rs1801253
rs7975232						rs4124874		rs2032582		rs1801272
						rs4149056		rs4124874		rs2066702
						rs701265		rs701265		rs28399499
						rs776746		rs776746		rs3745274
						rs7975232		rs7975232		rs3782905
										rs3815459
										rs4124874
										rs4148323
										rs4149056
										rs701265
										rs776746

**Fig. 2:**
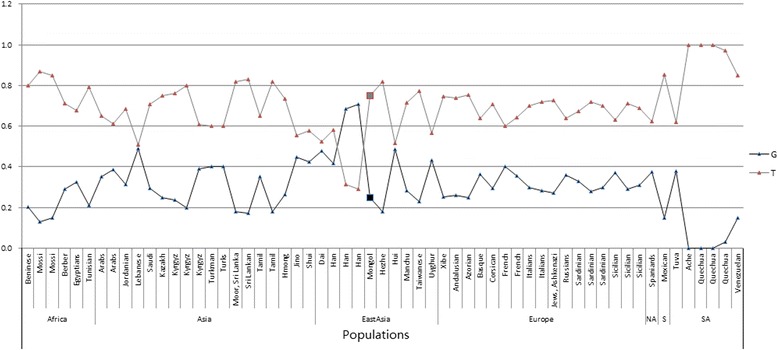
The global frequency distribution of rs1801131. NA, North America; SA, South America

**Fig. 3:**
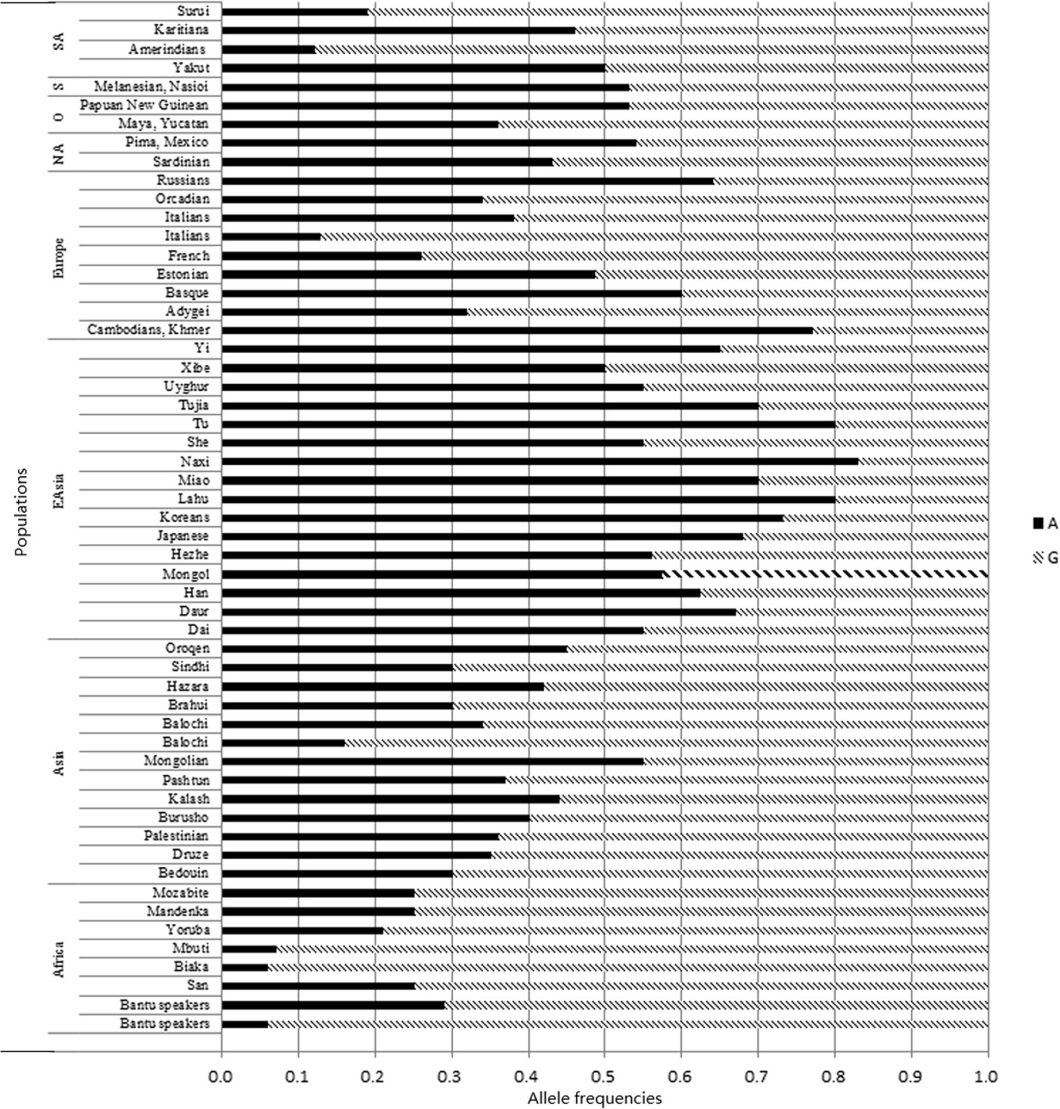
Rs1540339 frequencies in various global populations. EAsia, East Asia; NA, North America; SA, South America

To further verify the ubiquitous differences between different groups through research the difference of maximum and minimum of two SNPs, we selected two variants, the most significantly different variants -- rs1540339, rs1801131 which is one of the least significantly loci distributed in all 12 populations, and downloaded the population data from the ALFRED database. Combining the new data, we carried out a global analysis. Figure 2 shows the global frequency data of rs1801131 and Fig. 3, the rs1540339 data. From the two figures, we only found that the frequency of Mongol is relatively close to the populations distributed in East Asia.

**Fig. 4:**
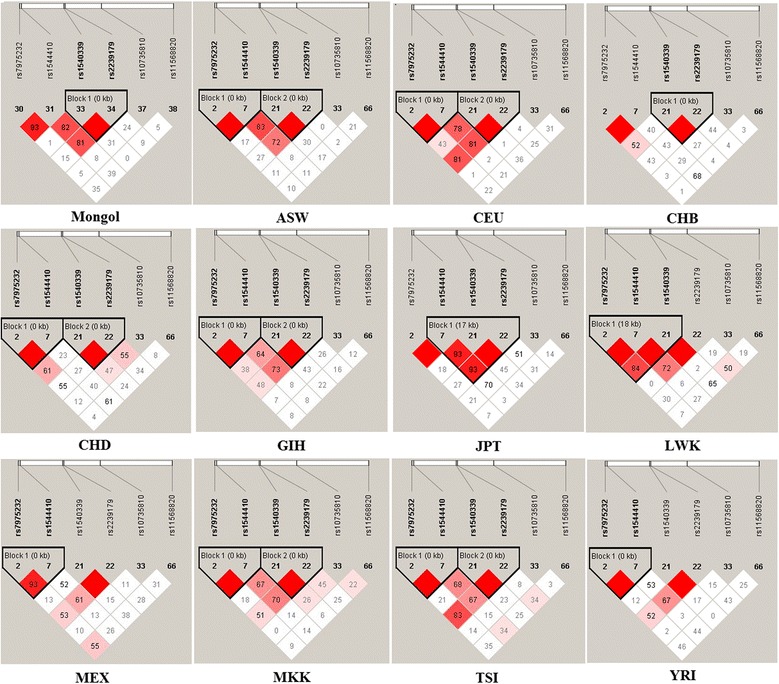
Linkage disequilibrium analysis of the VDR in each of the twelve populations. LD is displayed by standard color schemes with bright red for very strong LD (LOD > 2, D ′ =1), pink red (LOD > 2, D ′ <1), blue (LOD < 2, D ′ = 1) for intermediate LD, and white (LOD < 2, D′ <1) for no LD

Meanwhile, we focused on rs1540339 to explore the difference of the haplotypes. We performed the LD analysis to define blocks and haplotypes of VDR gene which include rs1540339, rs7975232, rs1544410, rs2239179, rs10735810 and rs11568820 by Haploview. The six SNPs selected from our lists and all of them have the HapMap data. Figure 4 shown that Mongol and CHB has only one block which consisted by rs1540339 and rs2239179, others has obviously different blocks compared with Mongol.

**Fig. 5:**
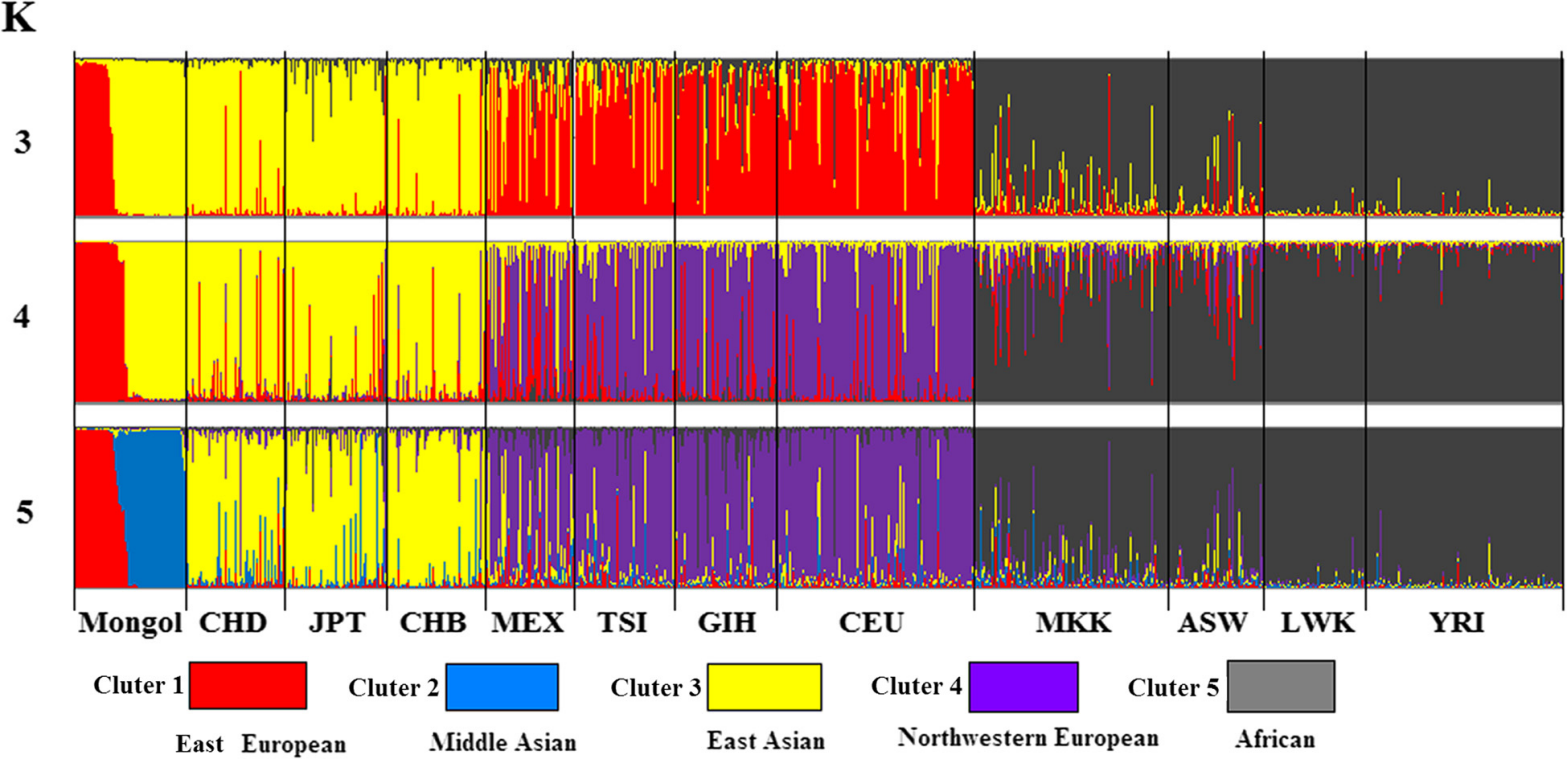
Structure analysis of the genetic relationship between 12 populations. K is the possible numbers of parental population clusters. One color represents one parental population cluster. Each individual is represented by a vertical column partitioned into different color segments. Most suitable K was observed at K = 5, where the proportion of each ancestral component in a single individual is represented by a vertical bar divided into 5 colors

For further clarified the genetic structure of Mongol and different populations, we used Structure 2.3.1 performed the population genetic structure comparisons by which works well for 85 loci (K = 2–8). The results are indicated by K = 3–5 (Fig. 5), which based on the Estimated Ln Prob of Data and other recommendations of the STRUCTURE software manual, When k = 3, individuals were divided in three affinity groups (subgroups 1: Mongol, CHD, JPT, CHB; subgroup 2: MEX, TSI, GIH, CEU; subgroup 3: MKK, ASW, LWK, YRI.) which used relative majority of likelihood assignment of individuals to subgroup. Followed by more K value to run STRUCTURE and then displayed the results in bar plots. From the image when k = 4 and 5, we easily found Mongol is closest to CHD, followed by CHB, JPT, and existed significant genetic structure differences with GIH and MEX.

## Discussion

Personalized or stratified healthcare is an important goal for medicine in the 21st century. It ensures that the treatments of patients are safe and efficacious [17]. With the rapid development of pharmacogenetics, serious attention has been paid to interethnic or interracial differences in drug responses with the intent to identify the genetic backgrounds of these variations [18]. Our study analyzed the distribution of these VIP variant allele and genotype frequencies to seek out which are altered among the different human populations [19], and found that even the SNP of smallest difference also had significant diversity between different groups. Through the comprehensive analysis, we revealed that Mongol and Chinese populations have the minimum difference.

Two of the variants were identified, rs1801133 (C677T) and rs1801131 (A1298C), included one of the least significant locus in our data, they are located in the same gene -- methylenetetrahydrofolate reductase (*MTHFR*) gene. *MTHFR* is located on chromosome 1p36.3 in human which is an important regulatory enzyme that involved in the folate pathway. It catalyzes the conversion of 5,10-methylenetetrahydrofolate to 5-methyltetrahydrofolate [20, 21]. Thymidylate synthesis required a lower 5,10-methylenetetrahydrofolate levels which leading to misincorporation of uracil into DNA, increasing chromosome damage frequency. A lower levels of 5-methyltetrahydrofolate may decrease the methylation process of homocysteine to methionine which could lead to hyperhomocysteinemia and DNA hypomethylation. Severe MTHFR enzyme deficiency is the most common inherited folate metabolism disorder which leads to hyperhomocysteinemia and homocystinuria that eventually destroy the central nervous system and vascular system [22]. Several studies revealed that the C677T and A1298C mutations reduce MTHFR enzyme activity [20–25]. In Caucasians, the C677T of TT and CT carriers had 70 % and 35 % reduced MTHFR enzyme activity, respectively, compared to CC carriers [26]. In Mongolians, CT and TT carriers had a frequency about 0.39 and 0.09. We should pay more attention on capecitabine, cisplatin, pemetrexed, cyanocobalamin and related agents in the Mongolian. Research of this mutation in other populations had not been performed. The enzyme activity reduction extent of different A1298C carriers had not been researched, but the study would play a large role in clinical treatment when one medication cure different patient who carriers different A1298C genotype.

We randomly selected one of the middle significantly different variants in Mongols -- the non-synonymous SNP rs1805124 (A1673G-H558R), which is located in exon 12 of *SCN5A* [27]. *SCN5A* encodes the integral membrane protein, voltage-dependent sodium channel α-subunit. It primarily traffics sodium in human heart muscle cells [28, 29]. SCN5A can cause fast depolarization during the upstroke phase of cardiac action potentials, that is the reason as a molecular antiarrhythmic drug target [30]. Amounts of Studies reveals SCN5A is associated with various cardiac diseases including long-QT syndrome (LQTS), Brugada syndrome (Brs), progressive cardiac conduction defect, atrial fibrillation (AF), dilated cardiomyopathy, and overlapping syndromes [27–31]. SCN5A-H558R has been shown to generate moderate electrophysiological functions that can regulate the phenotypic expression of cardiac conduction. It is associated with the mechanism of atrial fibrillation [30, 32] and can modify QTc duration in people with LQTS [33]. Studies of different genotype frequencies in various populations related to SCN5A-H558R function have not yet been performed, but SY Nikulina.et.al already found that AG genotype of the H558R (rs1805124) polymorphism of the SCN5A gene is a genetic predictor of idiopathic disorders of atrioventricular and intraventricular conduction [34] We can carry out the prevention and early treatment of these diseases by gene sequencing.

Among Mongols and others global populations, numerous important genetic variants play critical roles in drug response and this information should directly applied to clinical guidelines. For instance rs1540339 (46489G > A), the most significant locus in our data, is associated with bronchodilator responsiveness [35]. Studies have been performed on the correlation between asthma and rs1540339; however, evaluation of this polymorphism in a clinical setting is not yet routine [36, 37].

Beyond the genetic factor, we also determined that long-term survival in different environments affects genetic adaption. Environmental pressures shape genotype distributions towards specific functions, particularly in pharmacogenetic genes. Studies by Janha et al., Sabbagh et al., and Fuselli et al. directly demonstrated that the different genotype frequencies of CYP2C19, NAT2, and CYP2D6 significantly differed between populations race, subsistence modes, and dietary habits also play a role in the evolutionary trajectory [38–40].

## Conclusions

Different populations exists different genetic distribute frequencies. The drug dosage and usage of different genotype carriers is difference. Identifying genotype distribution and VIP variant frequencies in different populations to determine what medications might be most effective may provide a theoretical foundation for safe drug administration and improved curative effects. Besides, we figured out the minimum allele difference between Mongol and CDX. We also preliminary supplemented the pharmacogenomic data on the Mongol ethnic group and illustrated the differences between Mongols and other populations, and finally found Mongol and Chinese populations have the minimum difference. To the study, the sample size is relatively small and further investigation using a larger cohort of Mongols is needed to verify the generalizability of our results, and would be help us to establish a more reasonable and effective individualized treatment plan.

## Abbreviations

ALFRED, the ALlele FREquency Database; ASW, a population of African ancestry in the southwestern USA; CEU, a northwestern European population; CHB, the Han Chinese in Beijing, China; CHD, the population of metropolitan Denver, Colorado, USA; GIH, the Gujarati Indians in Houston, Texas, USA; HWE, Hardy–Weinberg Equilibrium; JPT, the Japanese population in Tokyo, Japan; LWK, the Chinese living in Luhya in Webuye, Kenya; MEX, people of Mexican ancestry living in Los Angeles, California, USA; MKK, the Maasai people in Kinyawa, Kenya; MTHFR, methylenetetrahydrofolate reductase; PharmGKB, the Pharmacogenomics Knowledge Base; PMT, the Pharmacogenetics of Membrane Transporters database; PUR, a population of Puerto Ricans from Puerto Rico; TSI, the Tuscan people of Italy; VIP, very important pharmacogenetic; YRI, the Yoruba in Ibadan, Nigeria
